# Optimal Timing for Intrauterine Device Insertion During the Menstrual Cycle: A Comparative Study of Pain, Bleeding, and Postprocedural Outcomes

**DOI:** 10.3390/clinpract16080139

**Published:** 2026-07-27

**Authors:** Lucian Șerbănescu, Sebastian Mirea, Vadym Rotar, Ștefan-Adrian Vrîncianu, Elena Mocanu, Maria Fulina, Stere Popescu, Cosmin Nișcoveanu, Radu-Andrei Baz

**Affiliations:** 1 Faculty of Medicine, Ovidius University, 900470 Constanta, Romania; rotarvadym.obst.gyn@gmail.com (V.R.); stere.popescu@365.univ-ovidius.ro (S.P.); cosmin_niscoveanu@yahoo.com (C.N.); raduandreibaz@yahoo.com (R.-A.B.); 2 County Clinical Emergency Hospital “St. Apostle Andrew”, 900591 Constanta, Romania; sebastian.mirea@umft.ro (S.M.); vrincianu.stefan@yahoo.com (Ș.-A.V.); maria.fulina@yahoo.com (M.F.); 3 Santerra Medical Center, 900178 Constanta, Romania

**Keywords:** intrauterine devices, contraception, intrauterine, device insertion timing, menstrual cycle phases, Pain Measurement, procedural bleeding, treatment outcome, patient tolerability

## Abstract

**Background:** Intrauterine device (IUD) insertion is a highly effective contraceptive procedure but is frequently associated with pain and bleeding, which may negatively influence patient acceptance and continuation rates. Although traditionally performed during menstruation, the optimal timing of insertion within the menstrual cycle remains uncertain, with limited data regarding patient-centered outcomes. **Objective:** The aim of this study was to evaluate the impact of menstrual cycle timing on pain, intra-procedural bleeding, and postprocedural outcomes in women undergoing IUD insertion. **Methods:** A comparative observational study was conducted including 200 parous women aged 20–40 years. Participants were consecutively enrolled and classified into two groups according to the day of the menstrual cycle at presentation for IUD insertion: Group A (days 3–7 of the menstrual cycle) and Group B (days 10–14). All procedures were performed by the same experienced gynecologist using a standardized technique. Pain was assessed using a Visual Analog Scale (VAS), intra-procedural bleeding was graded on a 0–3 scale, and postprocedural outcomes included analgesic use and bleeding characteristics. Statistical analysis was performed using independent t-tests and chi-square tests. **Results:** Baseline characteristics were comparable between groups (*p* > 0.05). Mean pain scores were significantly lower in Group B compared to Group A (3.1 ± 1.2 vs. 5.8 ± 1.4, *p* < 0.001). Intra-procedural bleeding was also significantly reduced in the mid-cycle group, with higher rates of minimal or no bleeding (85% vs. 20%, *p* < 0.001). The need for postprocedural anti-inflammatory medication was significantly lower in Group B (22% vs. 76%, *p* < 0.001). Additionally, postprocedural bleeding duration and intensity were reduced in the mid-cycle group. **Conclusions:** IUD insertion performed during the mid-cycle phase was associated with significantly better tolerability, including reduced pain, less bleeding, and decreased need for analgesia. While current guidelines allow flexible timing, these findings suggest that mid-cycle insertion may be associated with improved patient comfort when clinically feasible to improve patient experience without compromising access to contraception. Further randomized studies are warranted to confirm these results.

## 1. Introduction

Intrauterine devices (IUDs) represent one of the most effective and widely used methods of long-acting reversible contraception worldwide. Both copper-containing and levonorgestrel-releasing IUDs (LNG-IUDs) have demonstrated high efficacy, safety, and high continuation rates, making them a preferred contraceptive option across diverse populations and healthcare settings [1,2,3].

Despite their well-established benefits, IUD insertion remains a procedure frequently associated with patient discomfort, pain, and variable degrees of bleeding. These factors may negatively influence patient acceptance, increase anxiety related to the procedure, and ultimately impact continuation rates and overall satisfaction [1,2,3,4]. As a result, improving the tolerability of IUD insertion has become an important objective in contemporary gynecological practice.

Traditionally, IUD insertion has been recommended during menstruation, based on the assumption of easier cervical dilation, reduced risk of undetected pregnancy, and more favorable insertion conditions. However, this practice is not universally supported by strong evidence, and recent studies suggest that insertion outside the menstrual phase may be equally safe and, in some cases, better tolerated by patients. Nevertheless, data regarding the optimal timing of IUD insertion within the menstrual cycle remain limited and sometimes inconsistent.

Pain perception during IUD insertion is multifactorial, being influenced by cervical resistance, uterine contractility, parity, and individual pain sensitivity. In addition, hormonal fluctuations throughout the menstrual cycle may affect cervical softness and uterine responsiveness, potentially altering the patient’s experience during the procedure. Similarly, the degree of bleeding associated with insertion may vary depending on endometrial thickness and vascularization at different cycle phases, further supporting the hypothesis that timing may play a clinically relevant role [1,2,3,4].

Given the lack of consensus and the limited number of comparative studies addressing this issue, the present study aims to evaluate the influence of menstrual cycle timing on pain, bleeding, and postprocedural outcomes in women undergoing IUD insertion. Specifically, we compared insertions performed during the early menstrual phase (days 3–7) with those performed during the mid-cycle phase (days 10–14), under standardized procedural conditions.

## 2. Materials and Methods

### 2.1. Study Design and Setting

A comparative observational study was conducted including a total of 200 women who underwent intrauterine device (IUD) insertion in a single gynecological center. The study aimed to evaluate the impact of menstrual cycle timing on procedural pain, bleeding, and postprocedural outcomes.

### 2.2. Participants

The study population consisted of women aged between 20 and 40 years, all of whom had a history of prior childbirth (parous women). Nulliparous women were not included. Only parous women were enrolled to reduce variability related to cervical resistance and insertion difficulty, thereby allowing a more homogeneous assessment of the association between menstrual cycle timing and procedural outcomes. This selection criterion was intended to minimize confounding due to parity, although it limits the generalizability of the findings to nulliparous women.

Participants were enrolled consecutively as they presented for IUD insertion during the study period. Allocation to the study groups was not randomized. Instead, women were assigned according to the day of their menstrual cycle at the time of presentation. Women presenting between days 3 and 7 were included in Group A, whereas those presenting between days 10 and 14 were included in Group B.

### 2.3. Types of Intrauterine Devices

All patients received IUDs from the same manufacturer to ensure consistency. The distribution of IUD types was as follows:

**Group A:** 85 copper T 380 A IUDs (Pregna International Limited, Mumbai, India) and 15 Mirena levonorgestrel-releasing IUDs (Bayer AG, Leverkusen, Germany).

**Group B:** 84 copper T 380 A IUDs (Pregna International Limited, Mumbai, India) and 16 Mirena levonorgestrel-releasing IUDs (Bayer AG, Leverkusen, Germany).

The type of IUD was selected according to routine clinical indications and patient preference, independently of menstrual cycle timing. Because the distributions of copper-containing and levonorgestrel-releasing IUDs were nearly identical between the two study groups, device type was considered unlikely to confound the comparison of procedural outcomes.

### 2.4. Standardization of Procedure

To minimize operator-related and procedural variability, all insertions were performed by the same experienced gynecologist using a standardized technique.

The procedure followed identical steps in all cases:

1. Vaginal examination and insertion of an appropriately sized speculum (medium or large, depending on vaginal anatomy).
2. Vaginal and cervical antisepsis.
3. Cervical stabilization using a Pozzi forceps.
4. Uterine sounding (hysterometry).
5. IUD insertion according to standard clinical protocol.
6. Ultrasound verification of IUD position after insertion.

The same set of instruments was used for all procedures, including speculum, Pozzi forceps and uterine sound.

### 2.5. Outcome Measures

#### 2.5.1. Pain Assessment

Patients were asked to complete a pain assessment form 15 min after IUD insertion. Pain intensity was assessed using a 10-cm Visual Analog Scale (VAS), where 0 indicated “no pain” and 10 indicated “the worst pain imaginable”. Patients were instructed to rate the maximum pain experienced during the insertion procedure. The questionnaire was completed 15 min after the procedure to allow immediate post-procedural recording while minimizing recall bias.

No validated anxiety assessment instrument was administered before the procedure.

#### 2.5.2. Intra-Procedural Bleeding

Bleeding during IUD insertion was evaluated by the attending physician using a semi-quantitative scale ranging from 0 to 3:

- 0 = no bleeding.
- 1 = minimal bleeding.
- 2 = moderate bleeding.
- 3 = significant bleeding.

The assessment was based on clinical judgment and operator experience.

#### 2.5.3. Postprocedural Outcomes

Postprocedural outcomes included:

- Need for anti-inflammatory medication (within 1–2 days after insertion).
- Duration and intensity of vaginal bleeding following the procedure.

#### 2.5.4. Demographic Characteristics

The age range of participants was 20–40 years.

Parity distribution:

- **Group A:** 68% primiparous, 14% secundiparous, 10% tertiparous, and 8% multiparous.
- **Group B:** 72% primiparous, 12% secundiparous, 8% tertiparous, and 8% multiparous.

Ethnic distribution included approximately 80% Romanian patients, 10% Turkish-Tatar, and 10% other ethnic backgrounds.

### 2.6. Inclusion and Exclusion Criteria

#### 2.6.1. Inclusion Criteria

- Women aged 20–40 years.
- Parous patients.
- Regular menstrual cycles.
- Eligibility for IUD insertion.

#### 2.6.2. Exclusion Criteria

- Known hypersensitivity to IUD components.
- Active pelvic infection.
- Abnormal uterine bleeding of unknown origin.
- Uterine malformations.
- Pregnancy or suspected pregnancy.
- Contraindications to IUD use according to international guidelines.
- Nulliparous women.

### 2.7. Statistical Analysis

Continuous variables are presented as mean ± standard deviation (SD) and were compared between groups using the independent-samples *t*-test. Categorical variables are presented as frequencies and percentages and were compared using the chi-square test.

For the primary outcome (VAS pain score), the mean difference, 95% confidence interval (CI), and Cohen’s *d* effect size were additionally calculated to quantify the magnitude of the observed difference. Statistical significance was defined as a two-sided *p* value < 0.05.

No formal correction for multiple comparisons was applied because the analyses were prespecified and focused on a limited number of clinically relevant outcomes. Therefore, *p*-values should be interpreted together with the estimated effect sizes and confidence intervals.

No a priori sample size calculation was performed because this was an observational study. The sample size was determined by the number of eligible women who underwent IUD insertion during the study period and met the inclusion criteria.

## 3. Results

A total of 200 women were included in the study, with 100 participants in each group. Group A underwent intrauterine device (IUD) insertion during the early menstrual phase (days 3–7), while Group B underwent insertion during the mid-cycle phase (days 10–14). Correct IUD placement was confirmed by transvaginal ultrasound in all participants immediately after insertion.

### 3.1. Baseline Characteristics

The two groups were generally comparable in terms of demographic and clinical characteristics (Table 1).

### 3.2. Pain Assessment

Pain intensity measured using VAS showed significantly lower values in Group B (Table 2).

### 3.3. Intra-Procedural Bleeding

No or minimal intra-procedural bleeding (bleeding score 0–1) was observed more frequently in Group B than in Group A (85% vs. 20%; relative risk, 4.25; 95% CI, 2.84–6.36; *p* < 0.001) (Table 3).

### 3.4. Postprocedural Analgesia Requirement

The requirement for postprocedural anti-inflammatory medication was significantly lower in Group B than in Group A (22% vs. 76%; relative risk, 0.29; 95% CI, 0.20–0.42; *p* < 0.001) (Table 4).

### 3.5. Postprocedural Bleeding

The distribution of the duration and intensity of vaginal bleeding after IUD insertion is shown in Table 5.

### 3.6. Subgroup Analysis

No statistical differences were observed in terms of the intensity of bleeding and pain during the IUD insertion procedure between the two types of IUDs (Table 6).

## 4. Discussion

The present study evaluated the association between menstrual cycle timing and procedural outcomes following intrauterine device insertion. Women undergoing mid-cycle insertion experienced lower pain scores, less intra-procedural bleeding, and reduced postprocedural analgesic use than those undergoing insertion during the early menstrual phase. These findings suggest that timing within the menstrual cycle may be relevant to patient-centered procedural tolerability, although the observational design does not allow causal conclusions.

### 4.1. Comparison with Existing Literature

Current international guidelines support IUD insertion at any point during the menstrual cycle provided that pregnancy can be reasonably excluded, emphasizing timely access to contraception rather than a specific insertion window [1,2,4]. However, these recommendations are primarily based on contraceptive efficacy and safety outcomes rather than patient-centered measures such as procedural pain, bleeding, and tolerability. In the present study, women who underwent IUD insertion during the mid-cycle phase experienced lower pain scores, less intra-procedural bleeding, and reduced postprocedural analgesic use than women undergoing insertion during the early menstrual phase. These findings suggest that, although IUD insertion is considered safe throughout the menstrual cycle, the timing of insertion may influence the patient’s procedural experience. When scheduling is clinically feasible, mid-cycle insertion may therefore represent a simple strategy to improve procedural comfort without affecting contraceptive effectiveness. Nevertheless, prospective randomized studies are needed to confirm these findings.

### 4.2. Pathophysiological Interpretation

The lower pain scores and reduced intra-procedural bleeding observed during mid-cycle IUD insertion are biologically plausible. During the peri-ovulatory phase, rising estrogen levels promote cervical softening, increase cervical mucus permeability, and improve tissue compliance, thereby reducing cervical resistance during transcervical procedures [5,6,7,8]. Experimental studies have demonstrated cyclical changes in cervical viscoelasticity and epithelial permeability that may facilitate instrumentation during the estrogen-dominant phase [5,6,9]. In contrast, the menstrual phase is characterized by increased prostaglandin activity, endometrial shedding, and local inflammatory processes that may contribute to increased uterine contractility, pain perception, and bleeding during IUD insertion [10,11,12,13]. Together, these physiological changes provide a plausible explanation for the improved procedural tolerability observed during mid-cycle insertion. However, because most available evidence derives from studies of reproductive physiology rather than procedural outcomes, these mechanisms should be regarded as biologically plausible explanations rather than proof of a direct causal relationship [14].

### 4.3. Pharmacological Interventions

Several pharmacological strategies, particularly cervical priming with misoprostol, have been investigated to improve procedural ease and reduce pain during IUD insertion [15,16,17]. However, randomized controlled trials and systematic reviews have generally demonstrated limited or no clinically meaningful benefit in routine practice, while reporting a higher incidence of adverse effects, including abdominal pain, cramping, nausea, and vaginal bleeding [18,19,20]. Although some studies have suggested improved ease of insertion in selected populations, these findings have not been consistently replicated [18,19,20,21]. In contrast, the present study suggests that physiological variations associated with menstrual cycle timing may influence procedural tolerability without pharmacological intervention. Nevertheless, because of the observational design of our study, no direct comparison can be made between endogenous hormonal influences and pharmacological cervical priming.

### 4.4. Parity

Parity is a well-recognized determinant of pain during IUD insertion. Previous studies have consistently shown that nulliparous women experience higher pain scores and greater procedural difficulty than parous women [19,20,21]. These differences are thought to be related to increased cervical resistance and reduced cervical canal compliance and may require additional methods of anesthesia [18,19]. In the present study, only parous women were included to minimize variability related to cervical characteristics and to allow a more homogeneous assessment of the association between menstrual cycle timing and procedural outcomes. While this approach strengthens the internal validity of the study, it also limits the generalizability of the findings to nulliparous women, who may respond differently to IUD insertion [19,21].

### 4.5. Psychological Factors

Psychological factors, particularly preprocedural anxiety and pain expectation, are recognized determinants of pain perception during IUD insertion [22,23]. Higher anxiety levels have been associated with increased procedural pain and may also prolong the procedure [22,23]. In the present study, psychological variables were not formally assessed; therefore, their influence on pain perception cannot be excluded [24,25,26]. However, the consistency of the observed differences between the two menstrual cycle phases suggests that physiological factors related to hormonal status may also contribute to the observed differences. Future prospective studies should incorporate validated measures of preprocedural anxiety to better distinguish the relative contributions of psychological and physiological factors.

### 4.6. Complications

Although IUD insertion is considered a safe procedure, pain, vasovagal reactions, and abnormal bleeding remain the most common procedure-related adverse events and are important determinants of patient satisfaction and continuation of IUD use [2,18]. Serious complications, including uterine perforation, pelvic infection, and device expulsion, are uncommon but should always be considered during patient counseling and clinical follow-up [27,28,29,30,31,32]. Imaging assessment of the intrauterine device is essential for the detection of perforation related complications [33]. Patients with intrauterine devices who also have involuntary urine loss cannot benefit from pelvic muscle electrostimulation therapy, due to the main risk that the IUD could move during these procedures. Kegel exercises and balneotherapy remain the main effective recovery therapies for these patients [34,35]. In the present study, women undergoing mid-cycle insertion experienced significantly lower pain scores, less intra-procedural bleeding, and reduced postprocedural analgesic use than those undergoing insertion during the early menstrual phase. These findings suggest that optimizing the timing of IUD insertion may represent a simple, non-pharmacological strategy to improve procedural tolerability without compromising access to effective contraception.

### 4.7. Supporting Evidence from Other Intrauterine Procedures

Evidence from hysteroscopy and other transcervical procedures supports the concept that hormonal status influences cervical characteristics and procedural conditions. Diagnostic hysteroscopy is commonly performed during the early follicular phase to optimize visualization and minimize bleeding, indicating that menstrual cycle timing may affect procedural ease beyond IUD insertion [36]. Although these observations are indirect, they further support the biological plausibility of the present findings while highlighting the need for prospective studies specifically evaluating menstrual cycle timing during IUD insertion.

### 4.8. Clinical Implications

The findings of the present study have several relevant clinical implications for everyday gynecological practice. While current international guidelines support IUD insertion at any point in the menstrual cycle when pregnancy can be reasonably excluded, these recommendations primarily address safety and contraceptive efficacy rather than procedural tolerability.

Our results suggest that, when scheduling is flexible and clinical conditions allow, performing IUD insertion during the mid-cycle phase may be associated with greater patient comfort by reducing pain, bleeding, and the need for postprocedural analgesia. This represents a simple, non-invasive strategy that does not require additional resources or pharmacological interventions.

Importantly, optimizing the insertion experience may have broader implications beyond the procedure itself. Fear of pain remains a major barrier to the uptake of intrauterine contraception, and improving tolerability may contribute to increased patient acceptance, reduced procedural anxiety, and potentially higher continuation rates.

However, these findings should not be interpreted as a rationale to delay IUD insertion in situations where immediate contraception is required. Same-day insertion remains a priority in order to avoid missed opportunities for effective contraception. Therefore, a patient-centered approach is recommended, balancing accessibility with procedural optimization.

In this context, clinicians may consider discussing timing options with patients and, when feasible, offering mid-cycle insertion as a strategy to enhance comfort, while ensuring that access to timely contraception is not compromised.

### 4.9. Limitations

Several limitations of the present study should be acknowledged. First, the observational design may introduce potential selection bias, despite the comparable baseline characteristics between groups. Although efforts were made to standardize the procedure, including the use of a single experienced operator, unmeasured confounding factors cannot be entirely excluded.

Second, pain assessment was based on self-reported measures, which are inherently subjective and may be influenced by individual pain thresholds, psychological factors, and prior expectations. Although this reflects real-world clinical practice, it may introduce variability that is difficult to control.

Third, psychological variables such as anxiety, anticipated pain, and prior exposure to negative information were not formally assessed, despite evidence suggesting their significant impact on procedural pain perception. Their absence limits the ability to fully adjust for these factors in the interpretation of the results.

Although the distribution of copper-containing and levonorgestrel-releasing IUDs was well balanced between the study groups, the study was not specifically designed or powered to detect device-specific differences. Furthermore, because the indication for LNG-IUD insertion may differ from that for copper IUDs, residual confounding related to the underlying clinical indication cannot be completely excluded. Although subgroup analysis did not demonstrate significant differences in pain or bleeding according to IUD type, future prospective studies evaluating a single IUD type or stratifying analyses by device type would further strengthen the evidence. Because participants were allocated according to the timing of presentation rather than by randomization, selection bias and residual confounding cannot be completely excluded.

Bleeding was assessed by the treating physician using a semi-quantitative clinical scale without blinding. The fact that the assessment of the bleeding was done by the same experienced doctor, who performed all the IUD insertion procedures, reduces the risk of interpretation errors. Because the operator was aware of the timing of insertion, observer bias cannot be excluded. Future prospective studies should incorporate blinded outcome assessment or validated objective bleeding measures whenever feasible.

Because only parous women were included, the findings cannot be directly extrapolated to nulliparous women, who may experience different levels of pain and procedural difficulty during IUD insertion.

The absence of an a priori sample size calculation should be considered a limitation, although statistically significant differences were observed for the primary and secondary outcomes.

Because the study was designed to evaluate procedural outcomes under routine clinical practice, a validated anxiety questionnaire was not incorporated into the study protocol. Future prospective studies should include standardized measures of preprocedural anxiety to better evaluate its influence on pain perception.

Finally, the study was conducted in a single center, which may limit the external validity of the findings. Additionally, while the results suggest a significant association between menstrual cycle timing and procedural tolerability, causality cannot be definitively established, and further prospective randomized studies are needed to confirm these observations.

Despite these limitations, the consistency and magnitude of the observed differences across multiple outcomes strengthen the validity of the findings.

## 5. Conclusions

The findings of the present study suggest that the timing of intrauterine device insertion within the menstrual cycle is associated with differences in procedural tolerability. Insertion performed during the mid-cycle phase is associated with reduced pain, decreased intra-procedural bleeding, and a lower need for postprocedural analgesia compared to insertion during the early menstrual phase.

While current guidelines support flexible timing of IUD insertion, these results suggest that menstrual cycle phase may represent a simple and clinically relevant factor that can be considered to optimize patient comfort. Importantly, this approach does not require additional resources or pharmacological interventions.

Although these findings should not delay IUD insertion when immediate contraception is required, incorporating cycle timing into clinical decision-making, when feasible, may contribute to improved patient experience and increased acceptance of intrauterine contraception.

Further prospective and randomized studies are needed to confirm these observations and to better define the role of menstrual cycle timing in optimizing procedural outcomes.

## Figures and Tables

**Table 1:** Baseline Characteristics of Study Population.

Variable	Group A (Days 3–7) n = 100	Group B (Days 10–14) n = 100	*p*-Value
**Age** (**years**)	**29.4 ± 5.7**	**31.6 ± 5.2**	0.005
**Age groups**			
20–25	30%	32%	>0.05
26–32	45%	44%	>0.05
33–40	25%	24%	>0.05
**Parity**			
Primiparous	68%	72%	>0.05
Secundiparous	14%	12%	>0.05
Tertiparous	10%	8%	>0.05
Multiparous	8%	8%	>0.05
**Ethnicity**			
Romanian	80%	82%	>0.05
Turkish-Tatar	10%	8%	>0.05
Other	10%	10%	>0.05

Although the mean age differed significantly between groups (29.4 ± 5.7 vs. 31.6 ± 5.2 years; *p* = 0.005), the absolute difference was small (2.2 years) and was not considered clinically meaningful. No statistically significant differences were observed in age-group distribution, parity, or ethnicity, indicating that the groups were broadly comparable at baseline.

**Table 2:** Pain Scores (VAS).

Parameter	Group A	Group B	Mean Difference (95% CI)	Cohen’s d	*p*-Value
Mean VAS score	5.8 ± 1.4	3.1 ± 1.2	2.7 (2.34–3.06)	2.07	<0.001

Patients in the mid-cycle group reported significantly lower pain scores than those in the early menstrual phase (3.1 ± 1.2 vs. 5.8 ± 1.4). The mean difference was 2.7 VAS points (95% CI 2.34–3.06), corresponding to a very large effect size (Cohen’s d = 2.07; *p* < 0.001).

**Table 3:** Intra-procedural Bleeding Score Distribution.

Bleeding Score	Group A (%)	Group B (%)	*p*-Value
0 (none)	5%	40%	<0.001
1 (minimal)	15%	45%	<0.001
2 (moderate)	50%	10%	<0.001
3 (significant)	30%	5%	<0.001

**Table 4:** Need for Anti-inflammatory Medication.

Outcome	Group A	Group B	Relative Risk (95% CI)	*p*-Value
Required AINS	76%	22%	0.29 (0.20–0.42)	<0.001
No AINS needed	24%	78%	—	<0.001

**Table 5:** Postprocedural Bleeding Characteristics.

Parameter	Group A	Group B	*p*-Value
Duration > 24 h	Majority	Rare	<0.001
Mean duration	~36–48 h	~18 h	<0.001
Intensity	Moderate	Minimal	<0.001

**Table 6:** Outcomes by IUD Type.

Outcome	Copper IUD	LNG-IUD	*p*-Value
Mean VAS score	4.5 ± 1.8	4.4 ± 1.7	0.74
Mean intra-procedural bleeding score	1.2 ± 0.8	1.3 ± 0.7	0.68

## Data Availability

The original contributions presented in this study are included in the article. Further inquiries can be directed to the corresponding author.

## Abbreviations

The following abbreviations are used in this manuscript:

IUD	Intrauterine device
VAS	Visual Analog Scale
LNG-IUD	Levonorgestrel-releasing intrauterine device
ACOG	American College of Obstetricians and Gynecologists
